# Human P2Y_11_ Expression Level Affects Human P2X7 Receptor-Mediated Cell Death

**DOI:** 10.3389/fimmu.2018.01159

**Published:** 2018-06-08

**Authors:** Karin Dreisig, Louise Sund, Maja Wallentin Dommer, Nikolaj Pagh Kristensen, Kim Boddum, Rannveig Viste, Simon Fredholm, Niels Odum, Marja Jäättelä, Søren Skov, Birgitte R. Kornum

**Affiliations:** ^1^Molecular Sleep Laboratory, Department of Clinical Biochemistry, Rigshospitalet, Glostrup Hospital, Glostrup, Denmark; ^2^Norwegian Centre of Expertise for Neurodevelopmental Disorders and Hypersomnias (NevSom), Oslo University Hospital, Ullevål, Norway; ^3^Department of Immunology and Microbiology, University of Copenhagen, Copenhagen, Denmark; ^4^Cell Death and Metabolism Unit, Center for Autophagy, Recycling and Disease, Danish Cancer Society Research Center, Copenhagen, Denmark; ^5^Department of Veterinary Disease Biology, Faculty of Health and Medical Sciences, University of Copenhagen, Copenhagen, Denmark; ^6^Danish Center for Sleep Medicine, Department of Neurophysiology, Rigshospitalet, Glostrup Hospital, Glostrup, Denmark

**Keywords:** P2RY11, P2RX7, A740003, Fluo-4, cyclic adenosine monophosphate, purinergic, YO-PRO-1

## Abstract

Adenosine triphosphate (ATP) is known to induce cell death in T lymphocytes at high extracellular concentrations. CD4^+^ and CD8^+^ T lymphocytes have a differential response to ATP, which in mice is due to differences in the P2X7 receptor expression levels. By contrast, we observed that the difference in human CD4^+^ and CD8^+^ T lymphocyte response toward the synthetic ATP-analog BzATP is not explained by a difference in human P2X7 receptor expression. Rather, the BzATP-induced human P2X7 receptor response in naïve and immune-activated lymphocyte subtypes correlated with the expression of another ATP-binding receptor: the human P2Y_11_ receptor. In a recombinant expression system, the coexpression of the human P2Y_11_ receptor counteracted BzATP-induced human P2X7 receptor-driven lactate dehydrogenase release (a marker of cell death) and pore formation independent of calcium signaling. A mutated non-signaling human P2Y_11_ receptor had a similar human P2X7 receptor-inhibitory effect on pore formation, thus demonstrating that the human P2X7 receptor interference was not caused by human P2Y_11_ receptor signaling. In conclusion, we demonstrate an important species difference in the ATP-mediated cell death between mice and human cells and show that in human T lymphocytes, the expression of the human P2Y_11_ receptor correlates with human P2X7 receptor-driven cell death following BzATP stimulation.

## Introduction

The adenosine 5'-triphosphate (ATP)-binding receptor P2X7 has been shown to play an important role in the regulation of immune cells (1, 2). Binding of extracellular ATP allows the receptor to form a cation channel within milliseconds that among other things promotes formation and release of various cytokines from innate immune cells (3). At higher ATP concentrations, the P2X7 receptor displays the property of pore formation (4). P2X7 receptor pore formation occurs within seconds and results in changes in membrane permeability. This change in membrane permeability places the P2X7 receptor as an important mediator of the programmed cell death termed pyroptosis, which is characterized as an altruistic cell death where cells swell, burst, and die to release cellular content that attracts other immune cells to continue the fight against infections (5).

This type of P2X7 receptor-mediated cell death has been found important in mouse lymphocytes, where it regulates the homeostasis of T lymphocyte subpopulations (6). A lower ATP-induced calcium signaling has been seen in mouse CD8^+^ T lymphocytes compared to that in CD4^+^ T lymphocytes. This correlated with the level of P2X7 receptor expression (7). In another study, ATP resulted in the surface exposure of phosphatidylserine measured by annexin V staining in mouse CD4^+^ cells, but not in CD8^+^ cells. This effect correlated with the protein level of the P2X7 receptor (8).

In the present study, we found that CD4^+^ and CD8^+^ T lymphocytes isolated from human donors showed different P2X7 receptor activity similar to mouse T lymphocytes. This was seen despite similar P2X7 receptor levels in human CD4^+^ and CD8^+^ T lymphocytes. We hypothesized that this effect could instead be attributed to another ATP-sensing receptor: the P2Y_11_ receptor. This receptor is a G-protein-coupled receptor (GPCR), and stimulation of the receptor results in the activation of adenylyl cyclase and phosphatidylinositol pathways (9, 10). As this receptor is not present in mice cells, it offers an alternative mechanism for altering human immune cell death. The P2Y_11_ receptor plays a role in various immunomodulatory pathways (11), e.g., it has been found that low *P2RY11* gene expression correlates with an increased ATP-induced cell death in human natural killer cell and T lymphocytes (12) and the P2Y_11_ receptor delays apoptosis in human neutrophils (13). In CD4^+^, CD8^+^, and NK cells, stimulation with a P2Y_11_ receptor agonist NF546 rescues cells from ATP-induced cell death (12). This suggests that immune cell death in the presence of high ATP in humans is controlled not only by the level of the P2X7 receptor, as murine data suggest, but by the overall balance of ATP-sensing receptors including the P2Y_11_ receptor. The human P2Y_11_ receptor thus represents an important target in the regulation of human T lymphocytes. In this paper, we provide evidence that P2Y_11_ receptor inhibits P2X7 receptor pore formation but not calcium signaling which occurs independently of P2Y_11_ receptor signaling.

## Materials and Methods

### Lymphocyte Isolation

Blood from healthy donors was collected under informed written consent as approved by the ethical committee of Region Hovenstaden, Denmark, under license H-3-2013-054. Peripheral blood mononuclear cells (PBMCs) were isolated from buffy coats from healthy donors by gradient centrifugation in LymphoPrep (#1114740, Axis-Shield). Negative selection was carried out on fresh cells with RosetteSep (#15022, #15023, StemCell) or from frozen PBMCs using EasySep (#19052, #19053, StemCell). Frozen PBMCs were quickly thawed, resuspended in fresh medium, and rested for 2 h at 37°C before use. Cells were kept in RPMI-1640 (#BE12-702F, Lonza) + 10% fetal bovine serum (FBS) (#S0115, Biochrom). Freezing was done in additional 10% FBS and 10% DMSO.

### Immune Activation and Gene Expression Measurement

Transfected cells in 24 wells were harvested and cell pellets stored at −80°C before mRNA extraction and gene expression measurements. Primary RosetteSep isolated cells were maintained as 8 × 10^5^/mL with or without the addition of Dynabeads-Human T-Activator CD3/CD28 (#11161D, Gibco, Life Technologies). Cell pellets were collected and quickly frozen days 0–3 at −80°C.

mRNA was extracted by RNeasy Mini Kit (#74106, Qiagen). cDNA synthesis was carried out using TaqMan Reverse Transcription Reagents (#N8080234, Invitrogen, Life Technologies). qPCR gene expression was performed using TaqMan Universal PCR Master Mix (#4369016, Applied Biosystems, Life Technologies) with *ACTB* (β-actin) and *GAPDH* as housekeeping genes (list of primers shown in Table 1). Two separate primer/probe sets were used to analyze *P2RY11* expression in primary T lymphocytes and transfected cells, as the primer/probe set used for primary cells spanned the 3'-untranslated region of the gene, which was not present in the vector. *B2M* and *EEF1A1* genes were used as housekeeping genes because *GAPDH* and *ACTB* are not stable following immune activation (14, 15).

**Table 1:** Human TaqMan Gene Expression Assay primer/probes (#4331182, Life Technologies) showing target gene, the cell samples analyzed using the respective primer/probe sets, and their probe numbers.

Target gene	Cell sample	Probe number
*P2RY11*	HEK-293, GripTite	Hs01038858_m1
*P2RY11*	T lymphocytes	Hs00267414_s1
*P2RX7* (isoform A and B)	HEK-293, GripTite, T lymphocytes	Hs00175721_m1
*GAPDH*	HEK-293, GripTite, T lymphocytes	Hs99999905_m1
*ACTB* (β-actin)	HEK-293, GripTite, T lymphocytes	Hs99999903_m1
*B2M* (β2-microglobulin)	T lymphocytes	Hs00984230_m1
*EEF1A1*	T lymphocytes	Hs00265885_g1

### Western Blot

Cell pellets were snap-frozen in dry ice and stored at −80°C until later use. The cells were lysed in RIPA buffer (#856545, RegionH Pharmacy) supplemented with Triton X-100 for a concentration of 1% and cOmplete protease inhibitor was added (#11697498001, Roche Diagnostics). Samples were stored at −20°C until further use. Protein was denatured for 5 min at 95°C in 25% Laemmli buffer [0.25 M Tris-HCl (#839847, RegionH Pharmacy), 40% glycerol (#104092, Merck Millipore), 20% 2-mercaptoethanol (#M-6250, Sigma-Aldrich), 2% sodium dodecyl sulfate (SDS) (#L4390, Sigma-Aldrich), 0.01% bromophenol blue (#B-7021, Sigma-Aldrich)] before loading into RunBlue SDS Precast 4–20% in 12-well 10 × 10 cm gel (#NXG42012, Expedeon) and run in RunBlue SDS running buffer (#NXB50500, Expedeon). Protein was transferred onto an ethanol-activated Hypond-P membrane (#10600057, GE Healthcare Life Sciences) in transfer buffer [Tris-Gly buffer (#843856, RegionH Pharmacy), 20% ethanol, 0.1% SDS (#161-0416, BioRad)]. Blocking solution for unspecific binding was prepared as 2% Amersham ECL Prime Blocking Reagent (#RPN418V, GE Healthcare Life Sciences) in TBST buffer [TBS (#838571, RegionH Pharmacy), 0.1% Tween20], and membranes were blocked for 1 h at room temperature (RT) before incubation in primary rabbit anti-human P2X7 receptor (#ab109246, Abcam) or loading control diluted in TBST buffer with 0.4% blocking solution at 4°C overnight. Loading control was 1:5,000 mouse anti-β-actin (#A5441, Sigma-Aldrich) with a size of 42 kDa. On the following day, membranes were washed three times in TBST before shaking for 1 h at RT in 1:40,000 secondary ELC donkey HRP-linked anti-rabbit IgG (#NA934V, GE Healthcare Life Sciences) or 1:20,000 ELC sheep HRP-linked anti-mouse IgG (#NA931V, GE Healthcare Life Sciences) diluted in TBST. After washing, membranes were developed with Amersham ECL Select Western Blotting Detection Reagent (#RPN2235, GE Healthcare Life Sciences) for 5 min at RT and illuminated with chemiluminescence on LAS-4000 system.

Stripping and re-blotting were done with re-blot strong solution (#2504, Merck Millipore) for 15 min followed by blocking and staining with a primary rabbit anti-human P2Y_11_ receptor antibody (#ab183096, Abcam) as described above. This antibody has been found suitable for the detection of the P2Y_11_ receptor in Western blotting (16). Loading control staining with anti-β-actin was done analog to the above following another round of stripping of the membrane.

### Flow Cytometry

Peripheral blood mononuclear cells were thawed and rested as described above. 10^6^ cells were placed per tube and washed with phosphate buffered saline (PBS) (#L0615-500, Biowest), 400 g for 5 min at 4°C before staining with Fixable Viability Dye eFlouro 506 (#65-0866, eBioscience) for 20 min at RT. Cells were blocked for unspecific binding in PBS with 10% human serum (#H4522, Sigma-Aldrich) for 15 min on ice. Then, they were washed with PBS and incubated with combinations of extracellular anti-human CD3-BV421 (#563798, BD Bioscience), anti-human CD4-FITC (#11-0047-42, eBioscience), or CD8-PE-Cy7 (#344712, Nordic BioSite) for 30 min at 4°C. The cells were washed in PBS with 0.5% human serum before fixation and stained as previously described (16). Briefly, human P2Y_11_ receptor staining was performed on unpermeabilized cells with 0.1-µg Lightning Link (#705-0030, Innova Biosciences, UK) APC-conjugated 1:100 rabbit anti-human P2Y_11_ receptor antibody (#ab140878, Abcam) against the extracellular part for 30 min. We have previously tested this antibody and found it specific for the detection of human P2Y_11_ over P2Y_1_ and P2Y_2_ in transfected cells by flow cytometry (16). Flow cytometry was done on FACSverse (BD Biosciences, USA), and the FACSuite software (BD Biosciences, USA) was used for data analysis.

### Silencing RNA (siRNA) Transfection

Cells were transfected with siRNA against *P2RY11* (#L-005691-00-0005) and non-target control #1 (#D-001810-10-05, SMARTpool, ON-TARGETplus, Thermo Scientific) using 0.125 nmol on 2.0 × 10^6^ cells in an Amaxa Nucleofector (Lonza) as previously described (17). The cells were following transfection cultured in RPMI-1640 containing 2 mM l-glutamine and 100 mg/mL penicillin/streptomycin (Sigma-Aldrich). All cells were supplemented with 10% human serum (The Danish National Bloodbank, Denmark) and stimulated with Dynabeads-Human T-activator CD3/CD28 (#11131D, Thermo Scientific).

### Cell Lines

Human embryonic kidney (HEK-293) cells were maintained in culture medium: DMEM (#BE12-604F, Lonza) + 10% FBS + 0.5% p/s (#DE17-602E, Lonza) at 37°C, 5% CO_2_, and humidified air. GripTite 293 MSR cell line (a generous gift from Søren Gøgsig Faarup Rasmussen, University of Copenhagen, Denmark) is a genetically engineered HEK-293 line expressing the human macrophage scavenger receptor for better surface adherence. GripTite cells were maintained in culture medium supplemented with 1% non-essential amino acid (NEAA) (#M7145, Sigma-Aldrich) and 600 µg/mL antibiotic selection agent Geneticin (#10131-019, Gibco, Life Technologies) at 37°C, 5% CO_2_, and humidified air. Cells were passaged two to three times per week with trypsin (#L0930-100, Biowest) and versene (#15040-066, Gibco, Life Technologies).

### Plasmids Constructs and Transient Transfection

Vectors used for transfection were pcDNA3.1 (empty vector), eGFP, human *P2RX7* in a pcDNA3.1 backbone, human *P2RY11* (#EX-Z1416-M02, GeneCopoeia), and human *P2RY11*-mCherry (EX-Z1416-M56, GeneCopoeia). The C919T mutation was created in the *P2RY11*-mCherry vector using QuikChange Lightning Site-Directed Mutagenesis Kit (#210518, Agilent Technologies) following the manufacturer’s protocol. Mutation primers were F: 5'-ggcatgaggccccacatcacctggtag-3' and R: 5'-ctaccaggtgatgtggggcctcatgcc-3' (LGC Biosearch Technologies, Denmark). The mutated plasmid construct was sequenced at Eurofins Genomics Sequencing Department, Germany.

We used a reverse transfection protocol where the cells are seeding in wells during transfection or by seeding transfected cells into wells to control for transfection efficiency. Cotransfection of eGFP was included in the control group in some experiments or the application of a vector construct of P2Y_11_ receptor fused to mCherry to visualize successful transfection. The mCherry tag changed neither the calcium nor the cyclic adenosine monophosphate (cAMP) response of the P2Y_11_ receptor as shown in Figure S1A in Supplementary Material. Also, the gene expression levels in cells following transfection were measured to compare gene expression levels to rule out group-to-group variation (Figure S1B in Supplementary Material).

Transfection of cells for lactate dehydrogenase (LDH) and pore formation measurements was carried out by allowing the formation of DNA:Lipofectamine complexes in a medium without selection agent for 5 min. DNA:Lipofectamine mixture was dissolved to a final concentration of 0.5% Lipofectamine 2000 Reagent (#11668-019, Invitrogen, Life Technologies) and 1 µg DNA/mL. Cells were resuspended in this solution and seeded as 5 × 10^4 ^cells/well poly-d-lysine (#P6407, Sigma)-coated transparent or black clear bottom 96-well plate or 2.5 × 10^5 ^cells/well in poly-d-lysine-coated 24-well plate for gene expression. After a 5-h incubation, the medium was changed to culture medium and incubated overnight at 37°C, 5% CO_2_, and humidified air.

Cells for calcium imaging were cultured as 9.5 × 10^5 ^cells/well in a 6-well plate for 24 h. Lipofectamine and DNA were dissolved in DMEM + 10% FBS + 1% NEAA for a total of 2 µg DNA/well and 0.25% Lipofectamine, respectively, and allowed to transfect overnight. Cells were washed in PBS and loosened with versene and trypsin and cultured overnight as 3 × 10^5 ^cells/well in a black clear-bottomed poly-d-lysine-coated 96-well plate before further analysis.

### Cell Death by LDH Measurement

Cells were incubated with the synthetic ATP-analog 3'-O-(4-benzoyl)benzoyl-ATP (BzATP, #B6396-25MG, Sigma) dissolved in water, P2X7 receptor antagonist A740003 (#sc-291774, Santa Cruz Biotechnology) dissolved in ethanol, 4,4'-[carbonylbis(imino-3,1-(4-methyl-phenylene)-carbonylimino)]-bis(naphthalene-2,6-disulfonic acid) tetrasodium salt (NF340) dissolved in water (#3830/10, Tocris) or 1% Triton X-100 (#T9284-500 mL, Sigma-Aldrich) for 2 h before LDH measurement. EasySep-isolated lymphocytes at a concentration of 10^5^ cells/well were incubated for 2 h in round-bottomed 96-well plates with various concentrations of the abovementioned compounds before LDH measurement. LDH release was measured using an LDH colorimetric assay (#ab102526, Abcam). Absorbance for the culture medium without cells was subtracted from the values before calculating LDH release. All experiments included a nicotinamide adenine dinucleotide standard curve and a positive LDH control supplied with the kit.

### Pore Formation by YO-PRO-1 Dye Uptake

Twenty-four hours after medium change, transfected cells were washed once in dye buffer prepared as Hank’s Balanced Salt Solution (HBSS), (#14170112, Gibco, Life Technology) with 0.2% YO-PRO-1^®^ Iodide 491/509 (#Y3603, Life Technologies). Plates were read with NOVOStar (BMG LABTECH GmbH) microplate reader for every 2 min at 489/510 for 8 min before manually adding stimulatory compounds. The plate was then read for 64 min every 2 min. Pore formation was assessed by calculating the change in fluorescence intensity as *F*_max_ − *F*_min_.

### Calcium Response by Fluo-4

One day after seeding in 96-well plate, the cells were washed with a washing buffer (HBSS (#14065-049, Gibco), 10 mM HEPES (#15630-080, Gibco), 2 mM probenecid (#P8761, Sigma) (pH = 7.4)) and next incubated in a loading buffer (2 µM Fluo-4 AM (#F14201, Molecular Probes, in DMSO), 0.02% pluronic acid F-127 (#P2443, Sigma, in DMSO) in washing buffer) for 30 min. Cells were washed and allowed to rest for 10 min at 37°C in a washing buffer before automatic addition of BzATP. The signal was detected using the NOVOStar microplate reader system in a 2-min time span. For each data point, the maximal increase in the signal above baseline signal before BzATP addition was calculated. The resulting dose–response curves were normalized using baseline as 0% signal and signal at the highest dose as 100%. Next, the curve was fitted using a four-parameter fit of either a sigmoidal dose–response curve (one receptor) or a biphasic sigmoidal dose–response curve (two receptors). The fitted curves were finally used to calculate EC_50_. All calculations were performed using GraphPad Prism 7.

### cAMP Response by ELISA

Transfected cells in 96-well plates were lysed for 10 min in 0.1 M HCl. Debris was removed by centrifugation at ≥600 *g* 5 min. The supernatant was analyzed with cAMP ELISA colorimetric kit (#ADI-900-066, Enzo Life Sciences) according to manufacturer’s protocol. Briefly, the plate was prepared with 50 µL/well neutralizing reagent. Blue conjugate and yellow antibody were added to each well and incubated for 2 h on a plate shaker. The samples were washed three times in a washing buffer before the addition of substrate and incubation 1 h without shaking. The reaction was stopped and read at 405 nm. For each data point, cAMP concentration in pmol/mL was calculated on the basis of a standard curve. The resulting dose–response curve was fitted using a four-parameter fit of a sigmoidal dose–response curve using GraphPad Prism 7.

### Statistics

All statistical analyses were carried out in GraphPad Prism 7. Gene expression in CD4^+^ and CD8^+^ T lymphocytes was compared in a two-sided *t*-test. Gene expression changes in CD4^+^ T lymphocytes following *in vitro* immune activation were analyzed with one-way ANOVA and Dunnett’s *post hoc* testing against day 0. LDH activity in transfected CD8^+^ T lymphocytes was analyzed with one-way ANOVA and Dunnett’s *post hoc* testing. The mean values from individual donors or experimental runs were used for statistical analysis using two-way ANOVA with two groups *post hoc* tested using Sidak’s multiple comparison testing for each concentration. Multiple dose–response curves were compared with two-way ANOVA and Tukey’s multiple comparison *post hoc* testing.

## Results

### Differential Activity of the P2X7 Receptor in Human CD8^+^ and CD4^+^ T Lymphocytes

**Figure 1:**
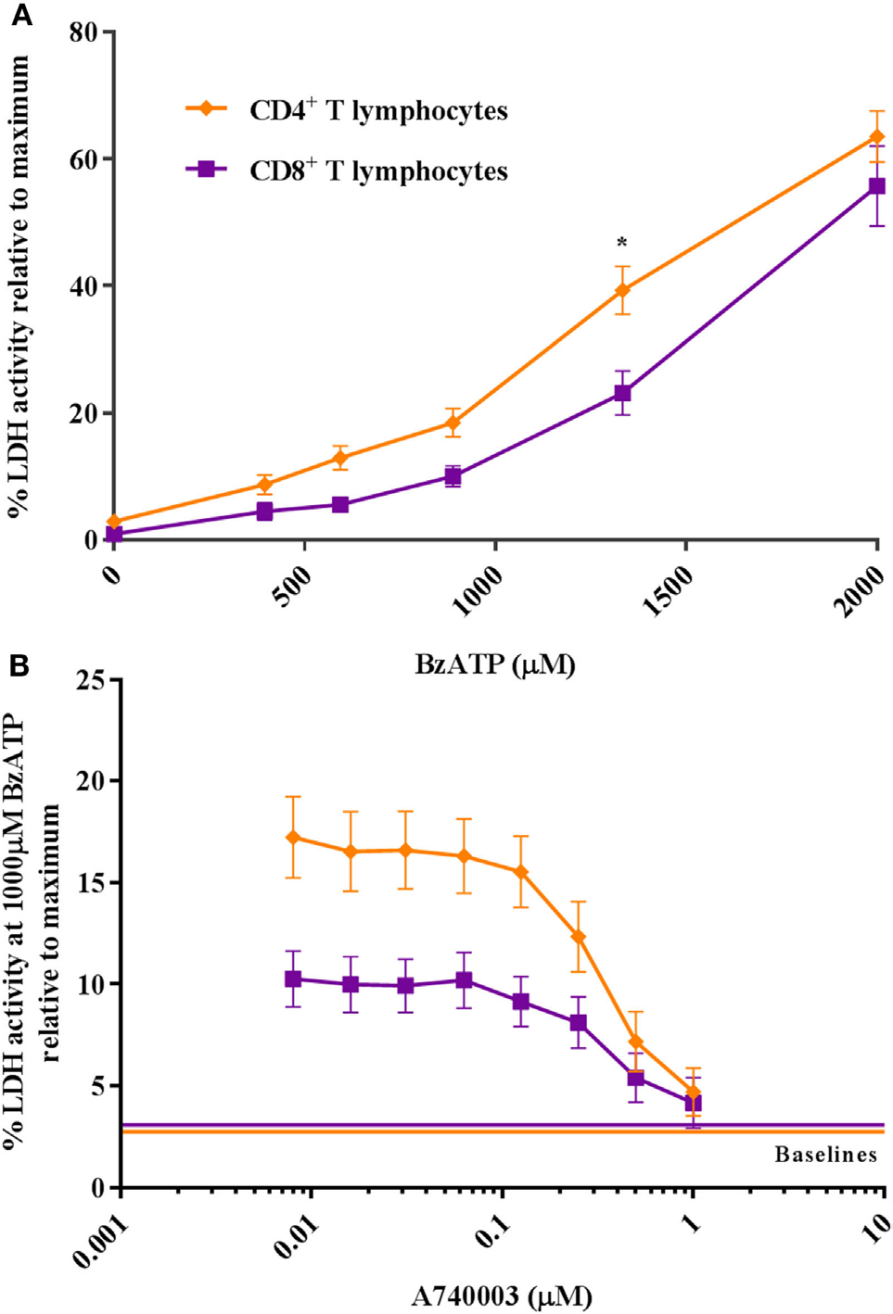
CD4^+^ and CD8^+^ T lymphocytes show a differential response to P2X7 stimulation. **(A)** The difference in CD4^+^ (orange rhomb) and CD8^+^ (purple square) T lymphocyte P2X7 receptor activation with BzATP was measured as lactate dehydrogenase (LDH) activity in the supernatant following 2 h stimulation. Data are shown as mean ± SEM normalized to maximum induced cell death in 1% Triton X-100, *n* = 5–6 from three independent donors. **(B)** The BzATP response in CD4^+^ and CD8^+^ T lymphocytes blocked by P2X7 receptor antagonist, A740003. Cells were stimulated with 1,000 µM BzATP in combination with various concentrations of P2X7 receptor antagonist, A740003, and P2X7 receptor activation was measured as the activity of LDH after 2 h. Data are shown as mean ± SEM of maximum induced cell death at 1% Triton X-100, *n* = 11–14 from seven independent donors. LDH baseline activity is shown as solid lines for CD4^+^ (orange) and CD8^+^ (purple) T lymphocytes, respectively.

To study the ATP-induced death of human CD4^+^ and CD8^+^ T lymphocytes, we treated cells isolated from healthy donors with increasing concentrations of the synthetic ATP-analog BzATP. Cell death was estimated by measuring LDH activity in the supernatant. This enzyme leaks from dying cells as a result of membrane permeabilization and can be used as an indirect indicator of plasma membrane integrity and cell death. The results showed that human CD4^+^ T lymphocytes released more LDH than CD8^+^ T lymphocytes (Figure 1A) after 2 h of incubation with BzATP. This difference is similar to what has been observed for CD4^+^ and CD8^+^ T lymphocytes in mice (7, 8). The increase in LDH release induced by BzATP could be completely blocked by the P2X7 receptor antagonist, A740003 (Figure 1B). Cells treated for 24 h showed similar levels of LDH release (Figure S2A in Supplementary Material), suggesting that cell death occurred during the first 2 h after BzATP stimulation. This is consistent with a previous report where P2X7 receptor-mediated cell lysis was observed as early as 15 min after stimulation with ATP in mouse lymphocytes (18).

### Similar P2X7 Receptor Expression in Human CD4^+^ and CD8^+^ T Lymphocytes

**Figure 2:**
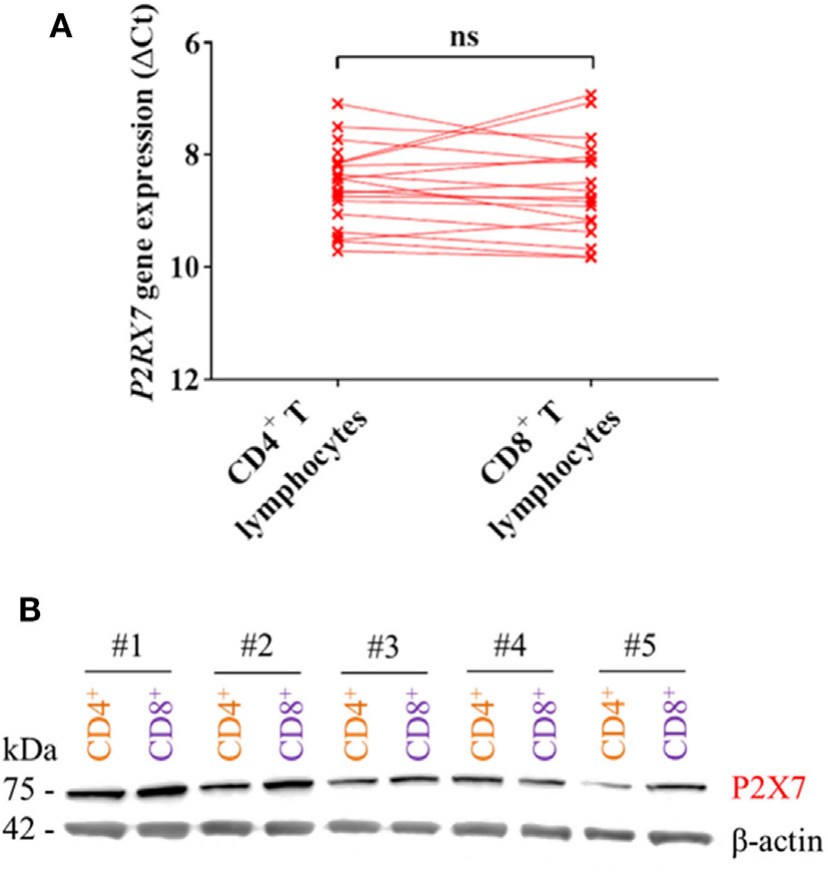
P2X7 levels are equally high in CD4^+^ and CD8^+^ T lymphocytes. **(A)** *P2RX7* gene expression in human CD4^+^ and CD8^+^ T lymphocytes shown as ΔCt values normalized to the genes for *GAPDH* and *ACTB* (β-actin) from 18 to 21 independent donors. **(B)** Western blot showing P2X7 receptor signal (75 kDa) from protein lysate of CD4^+^ (orange) and CD8^+^ (purple) T lymphocytes from five different donors (#1–5). Loading control was β-actin (42 kDa).

The higher P2X7 receptor-mediated response in mouse CD4^+^ T lymphocytes compared to CD8^+^ T lymphocytes has been explained by differences in expression levels of the P2X7 receptor (7, 8). Therefore, *P2RX7* gene and protein expression in human CD4^+^ and CD8^+^ T lymphocytes were measured. Surprisingly, there was no difference in the expression levels of *P2RX7* mRNA between human CD4^+^ and CD8^+^ T lymphocytes (Figure 2A), and the P2X7 receptor protein level was similar or slightly lower in CD4^+^ T lymphocytes compared to that in CD8^+^ T lymphocytes from five different human donors when examined by Western blot (Figure 2B). A similar human P2X7 receptor expression was previously been found in human CD4^+^ and CD8^+^ T lymphocytes with flow cytometry (19, 20). This shows that in human individuals, the greater P2X7 receptor-induced LDH release from CD4^+^ T lymphocytes following BzATP stimulation is not explained by higher P2X7 receptor expression levels.

**Figure 3:**
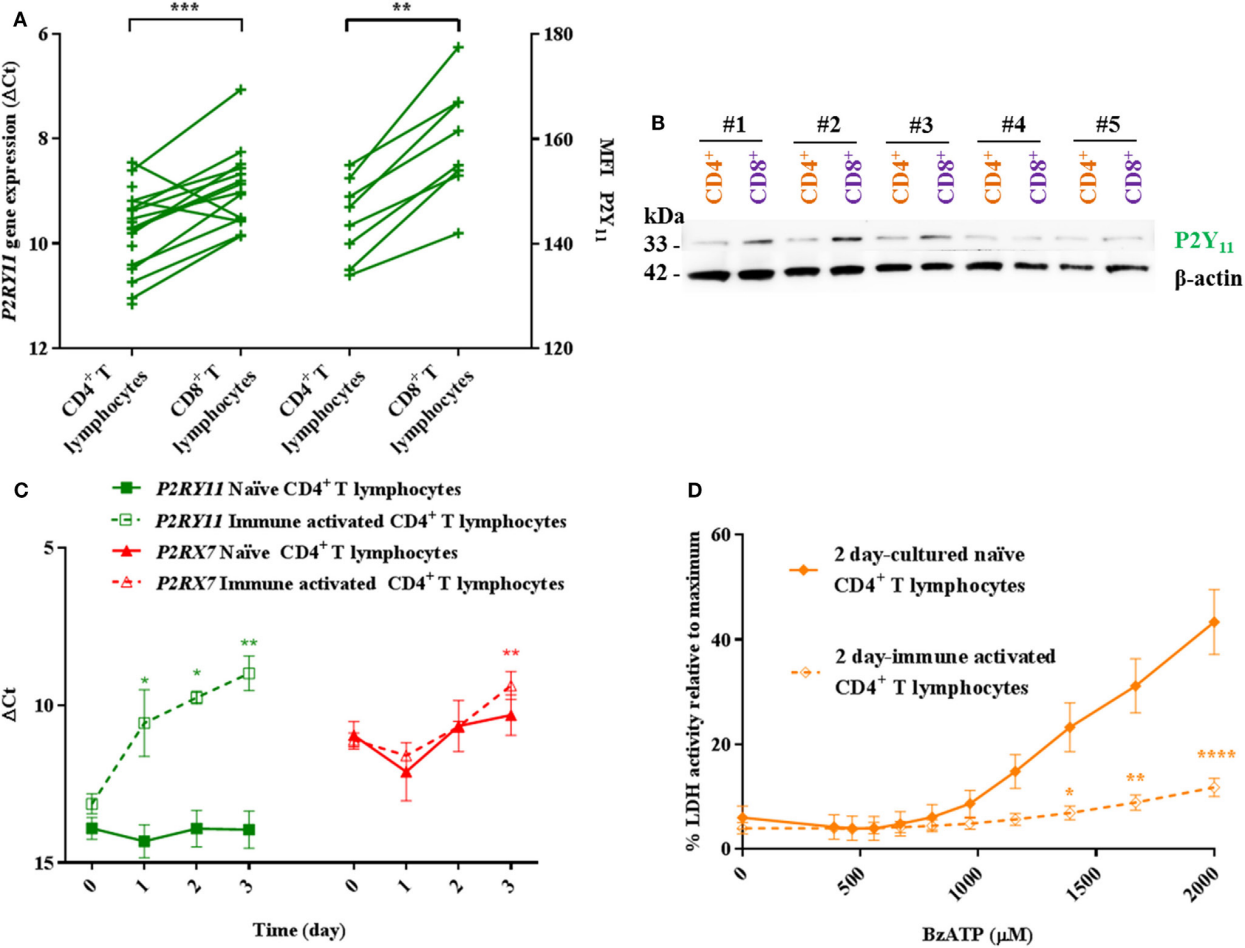
Higher P2Y_11_ levels in CD8^+^ T lymphocytes and immune-activated CD4^+^ T lymphocytes correlate with lower responses to BzATP compared to naïve CD4^+^ T lymphocytes. **(A)** Left axis: *P2RY11* gene expression in human CD4^+^ and CD8^+^ T lymphocytes shown as ΔCt values normalized to the genes for *GAPDH* and *ACTB* (β-actin) from 14 to 18 independent donors. Right axis: mean fluorescence intensity (MFI) as measured by flow cytometry on eight independent donors. **(B)** Western blot membrane with protein lysate of CD4^+^ (orange) and CD8^+^ (purple) T lymphocytes from five different donors (#1–5) following stripping and re-blotting for P2Y_11_ signal (33 kDa). Loading control was β-actin (42 kDa) after stripping and re-blotting. **(C)** ΔCt values for human *P2RY11* (green) and *P2RX7* (red) gene expression over time in naïve and immune-activated CD4^+^ T lymphocytes. Values were normalized to the genes for *B2M* and *EEF1A1* from three independent donors. **(D)** Lactate dehydrogenase (LDH) activity in the supernatant following 2 h incubation with various concentrations of BzATP of 2-day cultured naïve (filled rhomb) or immune-activated (open rhomb) CD4^+^ T lymphocytes. Data are shown as mean ± SEM of maximum induced cell death at 1% Triton X-100, *n* = 4–6 from three independent donors.

We, therefore, hypothesized that the differential BzATP response in human CD4^+^ and CD8^+^ T lymphocytes was due to a protective effect of a high P2Y_11_ receptor expression counteracting P2X7 receptor-mediated cell death in CD8^+^ T lymphocytes. This was based on previous findings that P2Y_11_ receptor stimulation mitigated the effect of ATP-induced cell death in PBMCs (12) and delayed apoptosis in neutrophils (13). The mRNA expression and protein levels of the P2Y_11_ receptor were indeed found to be significantly higher in human CD8^+^ compared to that in CD4^+^ T lymphocytes (Figures 3A,B) complying with the hypothesis.

### Immune Activation of Human CD4^+^ T Lymphocytes *In Vitro* Causes Upregulation of *P2RY11*

Immune-activated murine CD4^+^ T lymphocytes have lower levels of P2X7 receptor and are more resistant to P2X7 receptor-dependent cell death than their naïve counterparts (21). Therefore, the level of *P2RX7* and *P2RY11* gene expression in human CD4^+^ T lymphocytes following *in vitro* immune activation was investigated. Over a 3-day period, both *P2RX7* and *P2RY11* gene expression levels increased in CD4^+^ T lymphocytes following immune activation. No changes in *P2RY11* or *P2RX7* gene expression levels were found in CD8^+^ T lymphocytes following immune activation (data not shown). In contrast to murine CD4^+^ T lymphocytes, human CD4^+^ T lymphocytes did not downregulate *P2RX7* gene expression upon immune activation. Instead, a higher *P2RX7* level was observed (Figure 3C). Immune-activated CD4^+^ T lymphocytes showed a marked decrease in BzATP-induced LDH release compared to naïve cells when tested at day 2 (Figure 3D). Clearly, the reduced BzATP response in the human cells was not caused by a decrease in *P2RX7* expression as shown for murine cells. In line with our hypothesis, *P2RY11* gene expression was more than 10-fold upregulated in human CD4^+^ T lymphocytes following immune activation (Figure 3C), suggesting that in humans, the lower P2X7 receptor-mediated LDH release from immune-activated CD4^+^ T lymphocytes is caused by an increased P2Y_11_ receptor expression.

### P2Y_11_ Receptor Inhibition in Human T Lymphocytes

Currently, the most specific P2Y_11_ antagonist known is NF340 that blocks P2Y_11_ stimulation by BzATP in the dose range of 0.1–10 µM (22). If the P2Y_11_ signaling inhibited P2X7 activation, we expected that antagonizing the P2Y_11_ receptor with NF340 would increase LDH release. NF340 had, however, no effect on BzATP-induced P2X7 activity in primary CD4^+^ and CD8^+^ T lymphocytes when tested in doses ranging from 7.4 to 200 µM (Figure S2B in Supplementary Material) on a baseline of 1,000 µM BzATP. We worried that the high concentrations of BzATP applied to induce significant P2X7 activation would make it impossible to block P2Y_11_ activation with NF340 or any other pathway-interfering compound. Therefore, to test the effect of P2Y_11_ signaling on P2X7 cell death, we turned to the use of siRNA.

**Figure 4:**
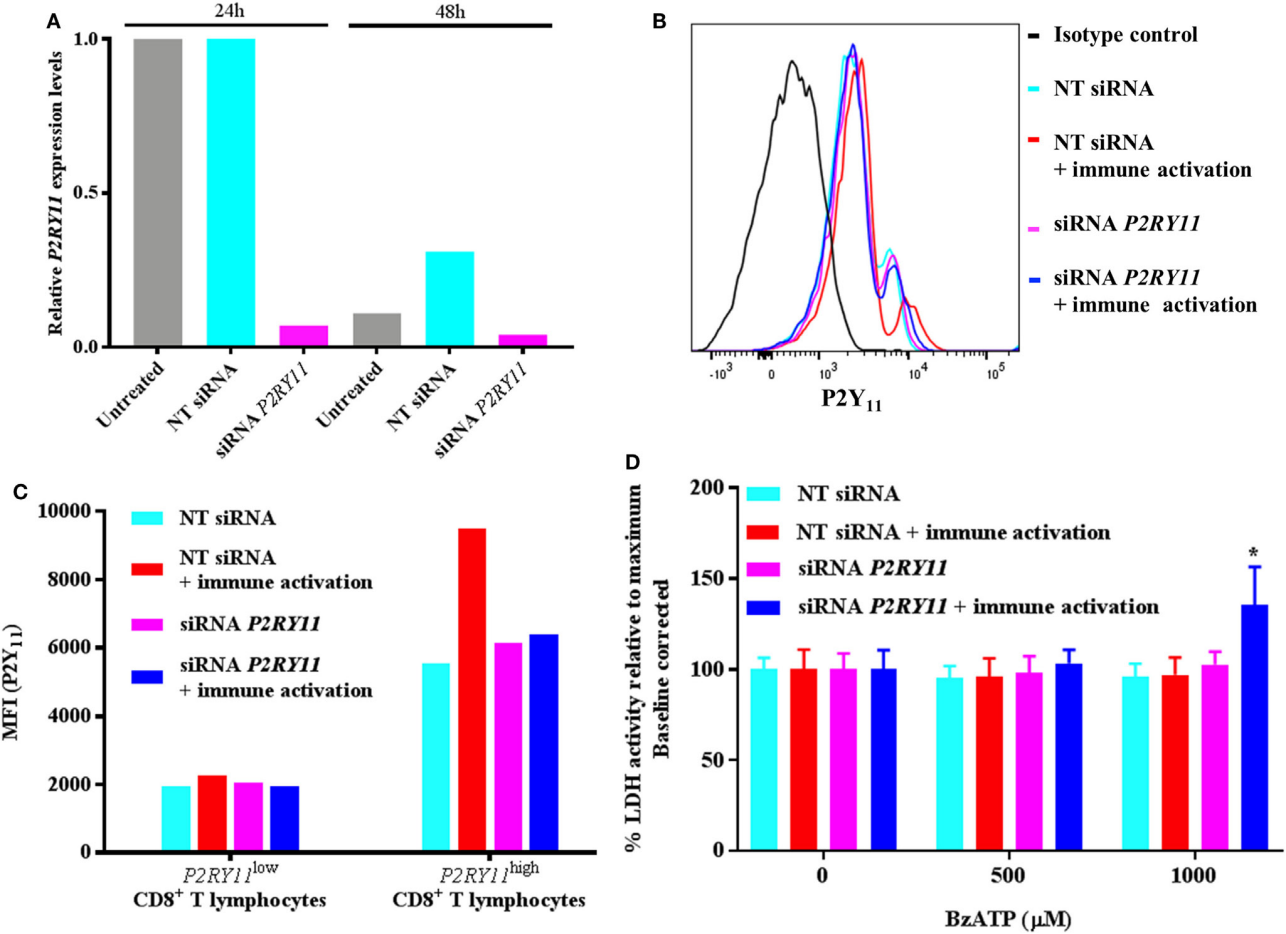
*P2RY11* silencing in CD8^+^ T lymphocytes shows increased BzATP-induced cell death following immune activation. **(A)** Relative *P2RY11* gene expression changes 24 and 48 h in CD8^+^ T lymphocytes left untreated (gray) or after transient transfection with non-targeting (NT) (turquoise) or siRNA targeting *P2RY11* (purple). **(B)** P2Y_11_ protein expression measured by flow cytometry count in isotype control (black), NT siRNA (turquoise), NT siRNA immune-activated (red), *P2RY11*-silenced (purple), or *P2RY11*-silenced immune-activated CD8^+^ T lymphocytes from a single donor. **(C)** Mean fluorescence intensity (MFI) from P2Y_11_ receptor protein expression measured by flow cytometry on transient transfected CD8^+^ T lymphocytes. **(D)** Lactate dehydrogenase (LDH) activity in transiently transfected CD8^+^ T lymphocytes stimulated 2 h with BzATP. Data are shown as mean ± SEM of maximum induced cell death at 1% Triton X-100 following baseline correction, *n* = 5–6 from two independent donors.

Transient transfection of CD8^+^ T lymphocytes with *P2RY11* siRNA resulted in an almost complete removal of *P2RY11* mRNA after 24 and 48 h (Figure 4A), but not in CD4^+^ T lymphocytes (data not shown). This decrease was not equally reflected in protein expression when examined with a flow cytometer. The P2Y_11_ receptor expression was close to identical between groups (Figures 4B,C). This suggested a low turnover of P2Y_11_ receptor on the CD8^+^ T lymphocytes. Having already found that immune activation could increase *P2RY11* gene expression in CD4^+^ T lymphocytes, we tested if immune activation upregulated P2Y_11_ receptor protein in the CD8^+^ T lymphocytes. The CD8^+^ T lymphocytes showed two populations with high and low P2Y_11_ receptor expressions, respectively (see gating strategy in Figure S3 in Supplementary Material). We found that the level of P2Y_11_ receptor protein was upregulated following immune activation in CD8^+^ T lymphocytes in the P2Y_11_^high^ subset (Figure 4C). This was not seen in immune-activated CD8^+^ T lymphocytes when *P2RY11* was silenced. Hence, silencing of *P2RY11* in human CD8^+^ T lymphocytes decreased *P2RY11* gene expression and prevented the cells from upregulating P2Y_11_ receptor protein expression following immune activation.

When the transfected CD8^+^ T lymphocytes were stimulated with BzATP, the immune-activated *P2RY11*-silenced CD8^+^ T lymphocytes had a significantly higher LDH release than immune-activated non-silenced cells (Figure 4D). This suggests that following immune activation, the T lymphocytes upregulate P2Y_11_ receptor, making them more resistant to cell death and that silencing *P2RY11* prevents the cells from using this protective mechanism.

### *P2RY11* Expression Counteracts P2X7 Receptor-Driven Cell Death

**Figure 5:**
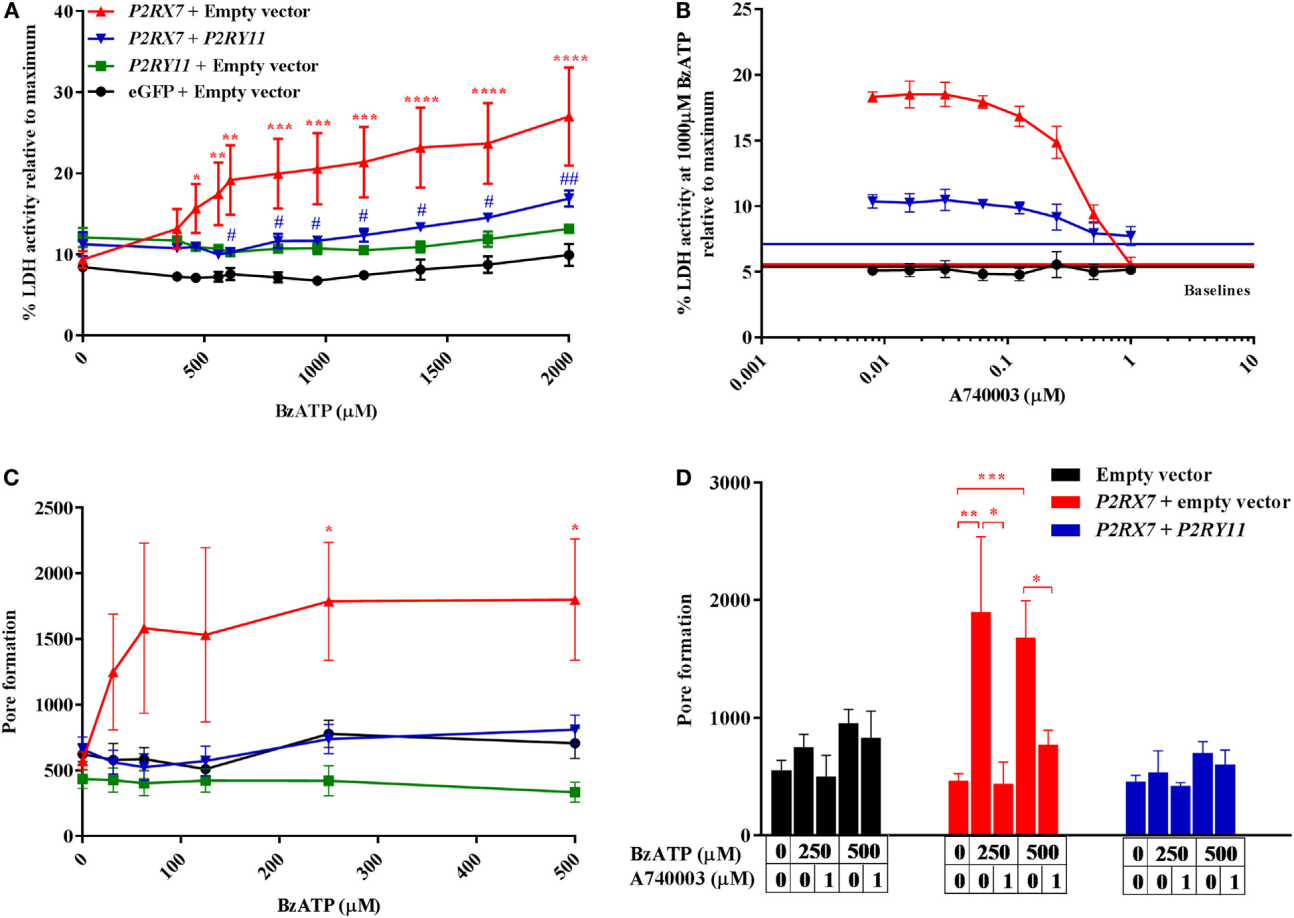
P2X7-mediated cell death and pore formation are lower when cells are cotransfected with *P2RY11*. Top row: the activity of lactate dehydrogenase (LDH) in the supernatant after 2 h in human embryonic kidney-293 (HEK-293) cells transiently transfected with vectors expressing purinergic receptors. Cells were stimulated with **(A)** increasing doses of BzATP, *n* = 5–6 from three experimental runs or **(B)** 1,000 µM BzATP in combination with various concentrations of P2X7 receptor antagonist, A740003, *n* = 6–8 from five experimental runs. Data are shown as mean ± SEM of maximum-induced cell death by 1% Triton X-100. LDH baseline activity is shown as solid lines for eGFP + empty vector (black), *P2RX7* + empty vector (red), and *P2RX7* + *P2RY11* (blue), respectively. Bottom row: GripTite cells transiently transfected with vectors expressing purinergic receptors were stimulated with various concentrations of BzATP to induce pore formation measured as YO-PRO-1 fluorescent dye uptake. Cells were stimulated with **(C)** increasing doses of BzATP, *n* = 12–29 from nine experimental runs or **(D)** BzATP and P2X7 receptor antagonist, A740003, *n* = 6–15 from five experimental runs. Data are shown as mean ± SEM.

Next, we studied the mechanism behind how P2Y_11_ receptor interfered with P2X7 receptor-driven membrane permeabilization and consecutive cell death in cell cultures overexpressing *P2RY11* and *P2RX7* separately or in combination. The cells were treated with various doses of BzATP, and P2X7 receptor-induced cell death was measured by LDH release into the supernatant after 2 h stimulation. As expected, cells overexpressing *P2RX7* displayed a significant increase in LDH release compared to control cells transfected with an empty vector (Figure 5A). This effect was mediated by the P2X7 receptor, as LDH release was completely inhibited by adding the P2X7 receptor antagonist A740003 (Figure 5B). Interestingly, coexpression of *P2RY11* prevented the BzATP-induced LDH release. This complies with our hypothesis that the P2Y_11_ receptor protects against P2X7 receptor-driven cell death. To rule out that the inhibition of LDH release was caused by a lower *P2RX7* expression in the cotransfected cells, the gene expression levels for *P2RX7* and *P2RY11* were measured in the transfected cells. Coexpression of *P2RY11* did not result in a decreased gene expression of *P2RX7* (Figure S1C in Supplementary Material).

### *P2RY11* Expression Counteracts P2X7 Receptor-Driven Pore Formation Independent of Calcium Signaling

Membrane permeabilization and LDH release as a response to ATP stimulation are believed to be caused by a pore-forming action of the P2X7 receptor (23). We, therefore, tested the hypothesis that *P2RY11* coexpression interfered with membrane permeabilization and LDH release observed in BzATP-treated cells by preventing P2X7 receptor pore formation. Cells overexpressing *P2RX7* were stimulated with BzATP, and YO-PRO-1 uptake was quantified as a measure of pore formation (Figure 5C). YO-PRO-1 enters the cell through the P2X7 receptor pore and becomes fluorescent upon DNA binding. A significant P2X7 receptor pore formation was observed at lower concentrations of BzATP than LDH release. The BzATP-induced increase in YO-PRO-1 signal was exclusively caused by dye uptake through the P2X7 receptor pore, as the P2X7 receptor antagonist A740003 completely removed the effect of BzATP (Figure 5D). At concentrations above 500 µM BzATP, the YO-PRO-1 dye signal from *P2RX7* transfected cells decreased (data not shown) most likely due to cell detachment following cell death. Coexpression of *P2RY11* completely inhibited pore formation in *P2RX7* expressing cells (Figure 5C).

**Figure 6:**
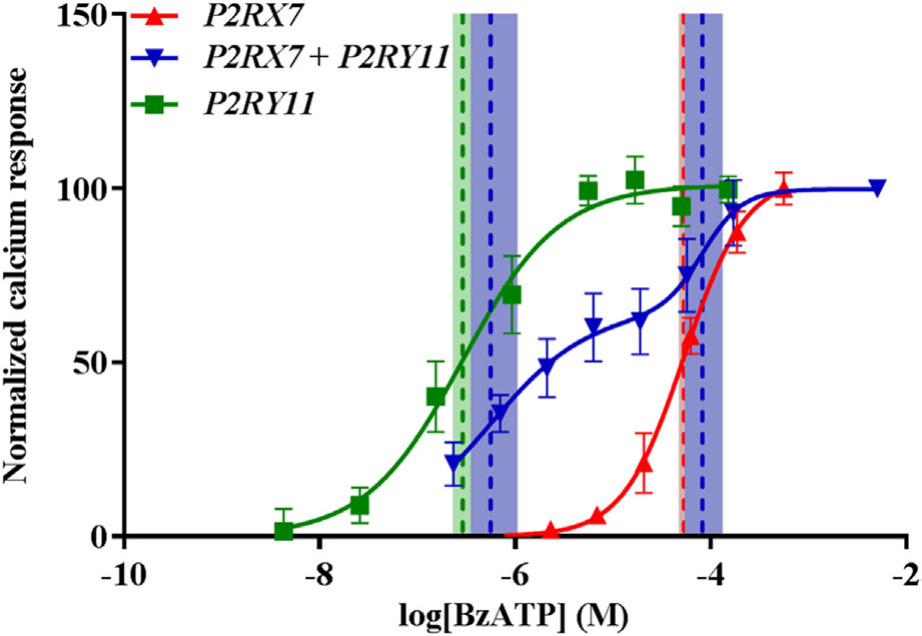
P2X7-mediated Ca^2+^ response is still present when cells are cotransfected with *P2RY11*. Normalized calcium response measured by Fluo-4 following stimulation with various concentrations of BzATP of transiently transfected GripTite cells. Data are shown as mean ± SD, *n* = 9 from three independent experiments. Dose–response curves for *P2RX7* (red) and *P2RY11* (green) were fitted to a sigmoidal curve and cotransfected *P2RX7* + *P2RY11* to a biphasic sigmoidal curve. Vertical dashed lines represent EC_50_ values for respective curves and 95% confidence intervals in colored shades. See text for details.

By contrast, calcium signaling through P2X7 receptor appeared unaffected as cells expressing both *P2RX7* and *P2RY11* showed a biphasic response to BzATP with EC_50_ values equal to their respective single-transfected counterparts (Figure 6). Values from fitted sigmoidal curves were *P2RY11* EC_50_ = 0.29 (0.23–0.36) μM and *P2RX7* EC_50_ = 53 (47–61) μM, respectively, and the values for the biphasic sigmoidal dose–response curve for *P2RX7* + *P2RY11* were EC_50_ = 0.56 (0.35–1.05) μM and EC_50_ = 82 (54–132) μM. This suggested that *P2RY11* expression interfered with P2X7 receptor pore formation independent of calcium signaling.

### *P2RY11*-Mediated Inhibition of P2X7 Receptor Dye Uptake Was Not Caused by P2Y_11_ Receptor Signaling

**Figure 7:**
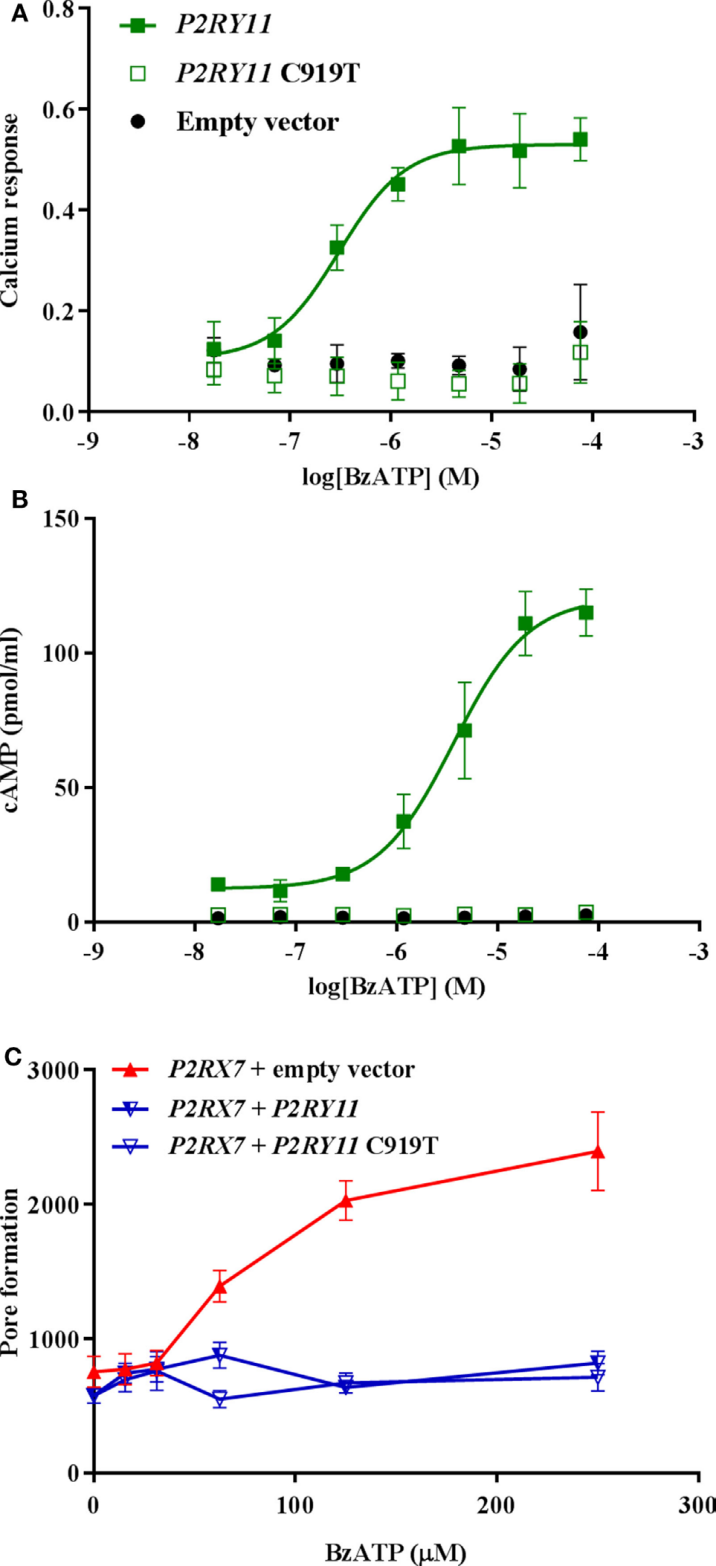
The effect of P2Y_11_ on P2X7-mediated pore formation is not dependent on P2Y_11_ signaling. Stimulation with various concentrations of BzATP of transiently transfected GripTite cells expressing empty vector (black), *P2RY11* (closed green square), or *P2RY11* C919T (open green square) vector in **(A)** calcium assay measured by Fluo-4, data are shown as mean ± SEM, *n* = 3,6 from 1 to 2 independent runs. **(B)** cAMP response, data are shown as mean ± SEM, *n* = 3,6 from 1 to 2 independent runs. **(C)** YO-PRO-1 dye uptake as a measure of P2X7 receptor pore formation in cells transfected with *P2RX7* + empty vector (red), *P2RX7* + *P2RY11* (half-open blue triangle), and *P2RX7* + *P2RY11* C919T (open blue triangle). Data are shown as mean ± SEM, *n* = 9 from three independent runs. Part of data was previously published by Ref. (25).

The P2Y_11_ receptor is a GPCR that signals *via* both G_s_ and G_q_ to activate the adenylyl cyclase and the phosphate inositol pathway (9, 10). We next asked whether the block of pore formation was caused by P2Y_11_ receptor signaling through G_s_ and/or G_q_ pathways. However, as the BzATP concentration needed to activate the P2X7 receptor at the same time saturated the P2Y_11_ receptor, it was not possible to activate P2X7 receptor while at the same time inhibiting P2Y_11_ receptor signaling (Figure 6). Instead, we tested a non-signaling P2Y_11_ receptor mutant in the cotransfection system. Among other amino acids, arginine 307 in the P2Y_11_ receptor has been predicted to be involved in ATP binding, interacting with the phosphate P_α_ moiety (24). This has been confirmed experimentally by mutational analysis, revealing inactivation of the receptor as measured by intracellular calcium release (24). Therefore, vectors expressing wild-type P2Y_11_ receptor and C919T-mutated *P2RY11* sequence resulting in the Arg^307^ being substituted with a tryptophan were tested. The mutated P2Y_11_ receptor was completely non-signaling as expected with regard to both calcium and cAMP signaling (Figures 7A,B). Coexpression of either P2Y_11_ or P2Y_11_ C919T receptors showed identical inhibition of P2X7 receptor YO-PRO-1 dye uptake, demonstrating that the P2Y_11_ receptor-induced inhibition of P2X7 receptor function is independent of signaling (Figure 7C).

## Discussion

This study provides evidence that human CD4^+^ and CD8^+^ T lymphocytes display differential responses to BzATP-induced P2X7 receptor activation similar to what has been seen for mouse lymphocytes. The difference in P2X7 receptor activity could not be explained by different levels of P2X7 receptor expression. This is consistent with previous studies showing identical surface P2X7 receptor expression levels in human lymphocyte subtypes from healthy donors (19, 20). Furthermore, we showed that *in vitro* immune-activated human CD4^+^ T lymphocytes did not significantly change *P2RX7* gene expression but that the cells nevertheless displayed a decreased LDH release following BzATP stimulation. By contrast, immune-activated CD4^+^ T lymphocytes showed a marked decrease in BzATP response. Clearly, human T lymphocytes have a different strategy for regulating P2X7 receptor activity than by transcriptional regulation of the *P2RX7* gene as in mice.

Instead, we hypothesized that another ATP-selective receptor, the P2Y_11_ receptor, regulates ATP response in human T lymphocytes. Indeed, *P2RY11* gene expression was significantly higher in human CD8^+^ T lymphocytes compared to that in CD4^+^ T lymphocytes consistent with the hypothesis that higher levels of the P2Y_11_ receptor reduce cell death following P2X7 receptor stimulation. We further observed that P2Y_11_ receptor was upregulated in T lymphocytes upon stimulation and that preventing this by siRNA treatment caused the immune-activated CD8^+^ T lymphocytes to become more vulnerable toward BzATP-induced cell death.

To investigate the possible interaction between the P2X7 receptor and P2Y_11_ receptor, a model system in HEK-293 cells transiently overexpressing the purinergic receptors was established. Two different end points for P2X7 receptor pore formation (LDH release and YO-PRO-1 dye uptake) were tested, and it was found that the presence of P2Y_11_ receptor inhibited P2X7 receptor pore formation in both assays; by contrast, P2Y_11_ receptor did not seem to interfere with calcium signaling following P2X7 receptor activation. This inhibition was not caused by downstream signaling from the P2Y_11_ receptor as demonstrated by using a loss-of-function mutant of the P2Y_11_ receptor.

Taken together, our results demonstrate that pore formation and cell death following P2X7 receptor activation are controlled by factors other than P2X7 receptor expression levels alone. One such factor is a P2Y_11_ receptor, which is particularly interesting as the P2Y_11_ receptor is also stimulated by ATP and BzATP. The difference observed in CD4^+^ and CD8^+^ T lymphocytes in cell death response following BzATP stimulation is therefore likely to be explained by differences in P2Y_11_ receptor expression levels and not by P2X7 receptor levels.

There are many possible ways in which *P2RY11* coexpression might interfere with P2X7 receptor pore formation. The exact mechanism for P2X7 receptor pore formation is still debated, but most literature is based on a two-conformation model in which the P2X7 receptor homo- or hetero-trimerizes upon ligand binding to form a cation channel and at long-lasting activation changes conformation into a larger pore (4). In our overexpression system, *P2RY11* in the transfected cells might block the normal processing and/or trafficking of the P2X7 receptor. P2Y_11_ receptor inhibition of P2X7 receptor surface expression might also occur in human lymphocytes that have been suggested to retain P2X7 receptor intracellularly under normal conditions (6, 26). Still, cells transfected with both *P2RX7* and *P2RY11* showed a biphasic response to BzATP in calcium-signaling assay showing that P2X7 receptor was present on the cell surface and capable of signaling as an ion channel.

The P2Y_11_ receptor might interfere directly with the P2X7 receptor through steric interaction in the membrane preventing pore formation. It could also indirectly interfere with other processes necessary for P2X7 receptor signaling such as pannexin-1 (27, 28) or P2× 4 receptor (4). Other G_q_-coupled P2Y receptor subtypes together with the P2Y_11_ receptor have been reported to interfere with ion channel function by interactions involving the C-terminal and the third transmembrane domain of the P2Y receptors (29, 30). This suggests that P2X7 receptor inhibition in human T lymphocytes could also be caused by other P2Y receptors. In retinal rat microvessels, the P2X7 receptor pore formation was found negligible because ATP also activated P2Y_4_ receptors and preventing P2X7 receptor pores from forming (31). It is possible that P2Y_11_ receptor in human T lymphocytes offers a species-specific mechanism for regulating immune cell death.

Likewise, P2Y_11_ receptors might also modulate other P2X receptors than the P2X7 receptor. It has, for instance, been shown that P2× 1 receptor signaling is changed when coexpressed with P2Y_1_ or P2Y_2_ receptors (32). Together, this is indicative of a complex network of interactions between purinergic receptors developed for fine-tuning of their many functions.

Regulation of ion channels through G-protein interaction is a well-documented phenomenon. An example is G-protein-activated inwardly rectifying K^+^ channels modulated by the G_βγ_ subunit (33). In addition, ion channels can also be indirectly modulated by second messengers such as seen for L-type calcium channels in the heart that are stimulated by cAMP (34). Cross-talk between ion channels and GPCRs sharing the same ligand has also been observed for GABA_B_ receptors (GPCR) that enhance GABA_A_ (ligand-gated ion channel) currents through a yet unidentified mechanism (35, 36).

In conclusion, our study demonstrates that the P2Y_11_ receptor is capable of interfering with P2X7 receptor pore formation and thus preventing cell death through a mechanism that does not involve signaling from the P2Y_11_ receptor. We suggest that this mechanism explains the decreased response in naïve human CD8^+^ T lymphocytes to extracellular ATP and that it also prevents P2X7-induced cell death in immune-activated CD4^+^ and CD8^+^ T lymphocytes.

## Ethics Statement

Blood from healthy donors was collected under informed written consent as approved by the ethical committee of Region Hovenstaden, Denmark, under license H-3-2013-054.

## Author Contributions

BK conceived and designed the study with valuable input from NO, MJ, and SS. LS, NK, and RV ran flow cytometry and KB assisted on calcium imaging studies. Western blot analysis was carried out by KD and MD. siRNA studies was carried out by SF. KD conducted all LDH and YO-PRO-1, gene expression, vector mutation, and cell culture transfections studies in addition to writing the manuscript with input from all authors.

## Conflict of Interest Statement

The authors declare that the research was conducted in the absence of any commercial or financial relationships that could be construed as a potential conflict of interest.

## Supplementary Material

Figure S1 **(A)** Normalized calcium (left axis) and cyclic adenosine monophosphate (cAMP) (right axis) responses in GripTite cells expressing *P2RY11* (filled square) and *P2RY11*-mCherry (half-open square), respectively, when stimulated with BzATP. Part of data was previously published (Degn et al., 2017, Brain) and is shown as mean ± SEM, *n* = 1–2. **(B)** Relative fold change in *P2RY11* (green) and *P2RX7* (red) gene expression from human embryonic kidney-293 (HEK-293) and GripTite cells transfected with vectors encoding the two purinergic receptors. Data are shown as mean ± range, *n* = 2–3. Relative fold changes were calculated using the ΔΔCt approach and genes for β-actin and GAPDH as housekeeping genes. Control represents eGFP + empty vector and empty vector-transfected HEK-293 and GripTite cells, respectively.

Figure S2 **(A)** Percentage of lactate dehydrogenase (LDH) activity in medium from CD4^+^ (orange rhomb) and CD8^+^ (purple square) T lymphocytes after 24 h stimulation with various concentrations of BzATP. Data are shown as mean ± SEM, *n* = 8–9 from three independent donors relative to maximum induced cell death by 1% Triton X-100. **(B)** Percentage of LDH activity in supernatant from CD4^+^ (orange rhomb) and CD8^+^ (purple square) T lymphocytes following incubation with 1,000 µM BzATP and P2Y_11_ antagonist, NF340. Data were normalized to maximum cell death induced by 1% Triton X-100 with control values labeled C. Data are shown as mean ± SEM from two independent donors.

Figure S3 Gating strategy for P2Y_11_ detection on CD8^+^ T lymphocytes by flow cytometry.
