# Supplementary figures and images for: Human P2Y11 Expression Level Affects Human P2X7 Receptor-Mediated Cell Death
Source: Front Immunol. 2018 Jun 8;9:1159. doi: 10.3389/fimmu.2018.01159 (PMC6002484; doi:10.3389/fimmu.2018.01159)

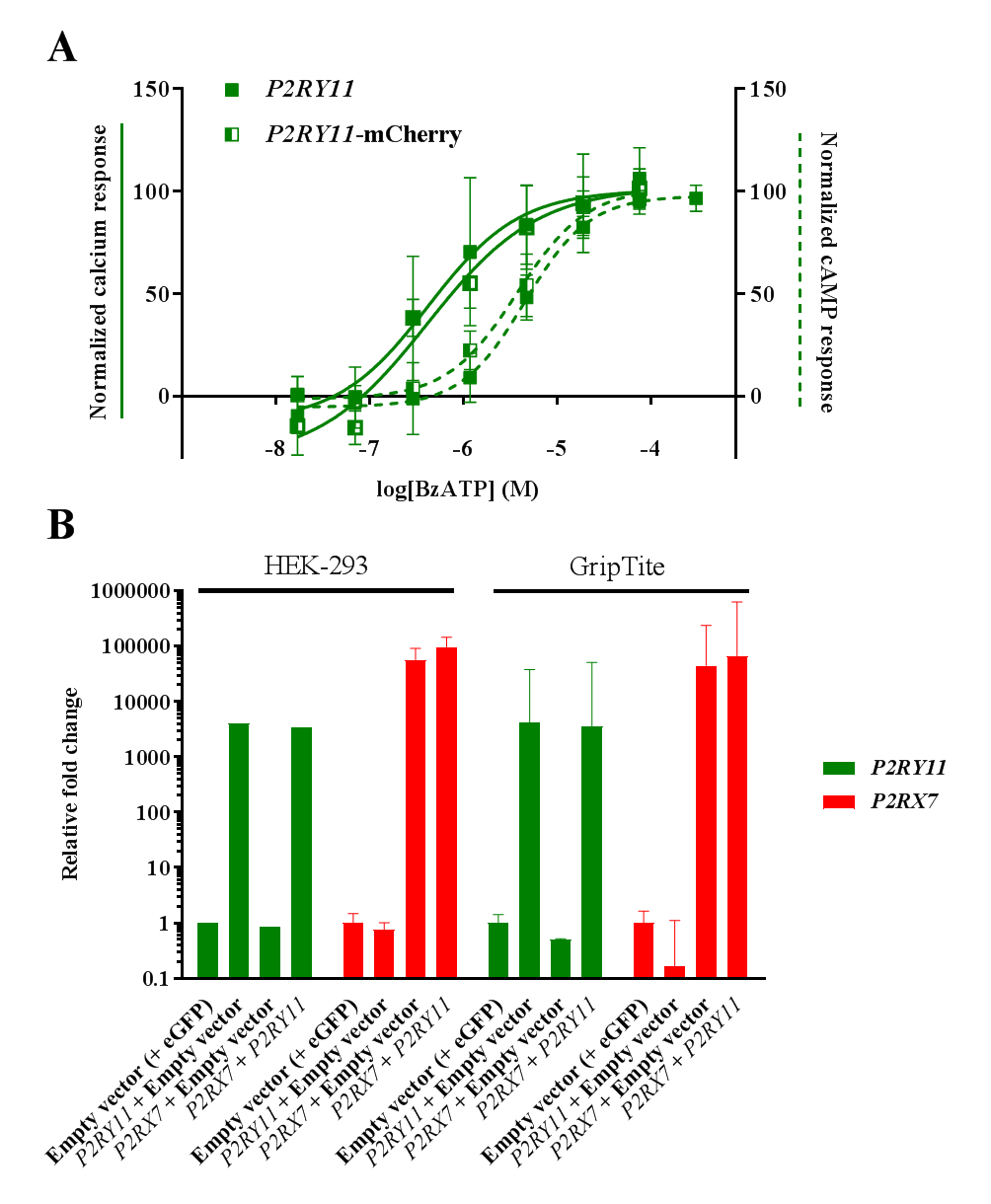

Supplement: Figure S1 — (A) Normalized calcium (left axis) and cyclic adenosine monophosphate (cAMP) (right axis) responses in GripTite cells expressing P2RY11 (filled square) and P2RY11-mCherry (half-open square), respectively, when stimulated with BzATP. Part of data was previously published (Degn et al., 2017, Brain) and is shown as mean ± SEM, n = 1–2. (B) Relative fold change in P2RY11 (green) and P2RX7 (red) gene expression from human embryonic kidney-293 (HEK-293) and GripTite cells transfected with vectors encoding the two purinergic receptors. Data are shown as mean ± range, n = 2–3. Relative fold changes were calculated using the ΔΔCt approach and genes for β-actin and GAPDH as housekeeping genes. Control represents eGFP + empty vector and empty vector-transfected HEK-293 and GripTite cells, respectively. [file image_1.tif]

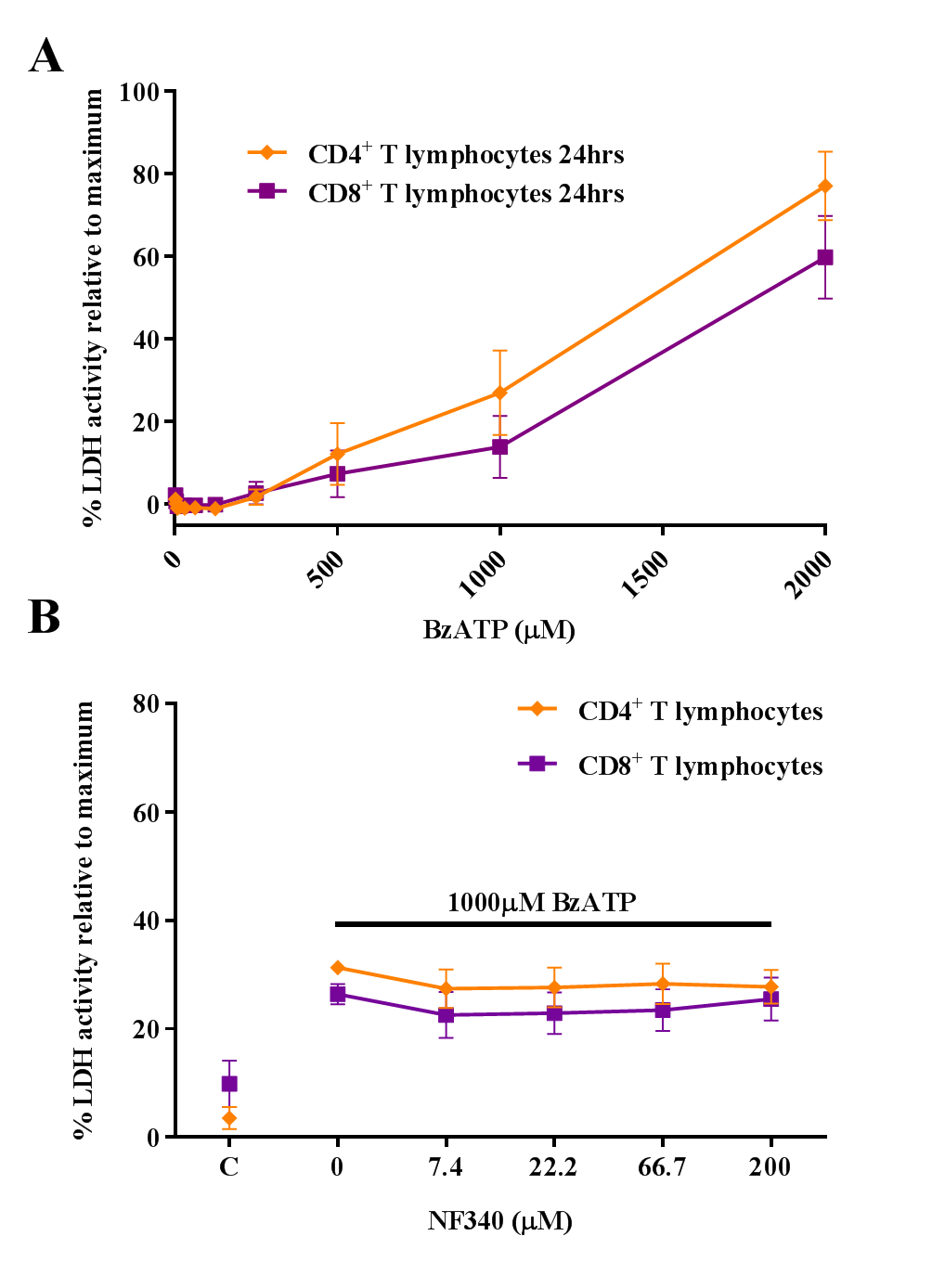

Supplement: Figure S2 — (A) Percentage of lactate dehydrogenase (LDH) activity in medium from CD4+ (orange rhomb) and CD8+ (purple square) T lymphocytes after 24 h stimulation with various concentrations of BzATP. Data are shown as mean ± SEM, n = 8–9 from three independent donors relative to maximum induced cell death by 1% Triton X-100. (B) Percentage of LDH activity in supernatant from CD4+ (orange rhomb) and CD8+ (purple square) T lymphocytes following incubation with 1,000 µM BzATP and P2Y11 antagonist, NF340. Data were normalized to maximum cell death induced by 1% Triton X-100 with control values labeled C. Data are shown as mean ± SEM from two independent donors. [file image_2.tif]

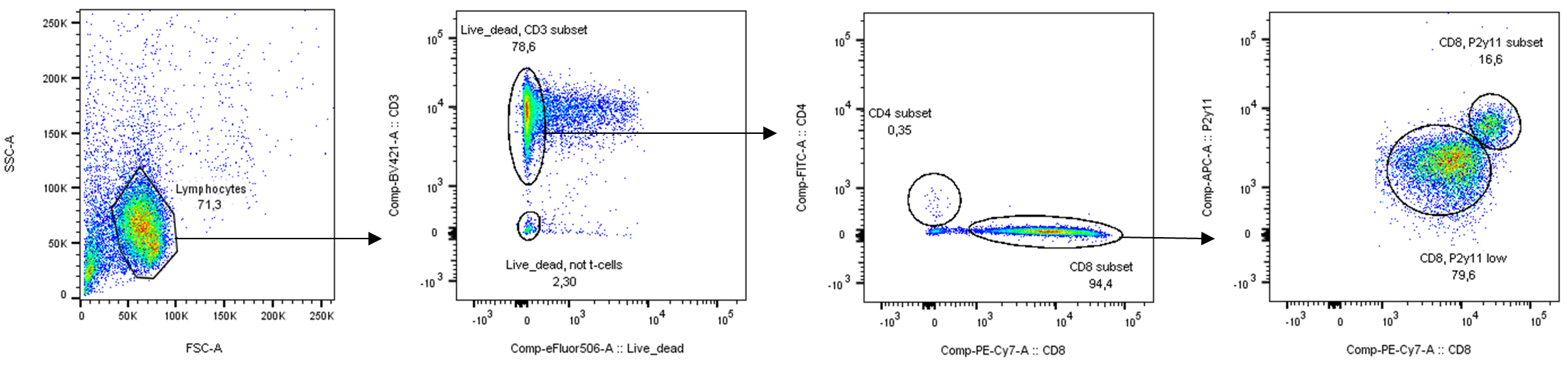

Supplement: Figure S3 — Gating strategy for P2Y11 detection on CD8+ T lymphocytes by flow cytometry. [file image_3.tif]
